# Evaluation of the Association Between Retinal Vein Occlusion and the Risk of Atrial Fibrillation Development: A 12-Year, Retrospective Nationwide Cohort Study

**DOI:** 10.1038/srep34708

**Published:** 2016-11-07

**Authors:** Tyler Hyungtaek Rim, Jaewon Oh, Christopher Seungkyu Lee, Sung Chul Lee, Seok-Min Kang, Sung Soo Kim

**Affiliations:** 1 Department of Ophthalmology, National Health Insurance Service Ilsan Hospital, Korea; 2 Division of Cardiology, Severance Cardiovascular Hospital and Cardiovascular Research Institute, Yonsei University College of Medicine, Seoul, Korea; 3 Department of Ophthalmology, Severance Hospital, Institute of Vision Research, Yonsei University College of Medicine, Seoul, Korea; 4 Yonsei Healthcare Big Data Based Knowledge Integration System Research Center, Yonsei University College of Medicine, Seoul, Korea; 5 Institute of Convergence Science, Yonsei University College of Medicine, Seoul, Korea

## Abstract

We aimed to evaluate the risk of atrial fibrillation (AF) development following retinal vein occlusion (RVO). We performed a nationwide propensity score-matched cohort study by retrospectively reviewing a database from the Korean National Health Insurance Service, comprising approximately 1 million random subjects. RVO and AF were diagnosed based on the Korean Classification of Disease codes. The RVO group was composed of patients with an initial diagnosis of RVO made between 2003 and 2007 (n = 1,801), excluding those who were diagnosed in 2002. The comparison group was composed of randomly selected patients (5 for each patient with RVO, n = 8,930) who were matched to the RVO group according to sociodemographic factors and the year of enrollment. Each sampled patient was tracked until 2013. The predictive value of RVO for AF was analyzed using Cox regression analysis with a hazard ratio (HR) and confidence interval (CI). AF developed in 6.5% of patients in the RVO group and 4.0% of those in the comparison group (p < 0.001). RVO was associated with a greater risk of AF development after adjusting for possible confounders (HR, 1.35; 95% CI, 1.09–1.67). An association between RVO and subsequent AF development was found after adjusting for possible confounding factors.

If the central retinal vein or one of its main four branches is occluded, retinal hemorrhage and retinal edema subsequently occur and frequently cause low vision or blindness in adults^1^. This is called retinal vein occlusion (RVO), and there are two types: branch RVO (BRVO) and central RVO (CRVO). The suggested mechanisms of CRVO are increasing intraocular pressure, an atherosclerotic central retinal artery, and deformation of the lamina. For BRVO, the suggested mechanisms are interrupted venous flow at the arteriovenous crossing by arterial stiffening due to atherosclerosis^2^. Several previous studies have shown that RVO increases the risk of cardiovascular/cerebrovascular disease and their associated mortalities^3^,^4^,^5^,^6^,^7^,^8^,^9^,^10^,^11^,^12^,^13^,^14^. Considering the data for the risks of subsequent stroke or myocardial infarction following RVO development, it seems likely that small-sized to medium-sized vascular disease of the eye is associated with a higher likelihood of cardiovascular disease.

Atrial fibrillation (AF) is common in the elderly, and it is a significant public health problem associated with increased mortality and high cardiovascular morbidities such as stroke and heart failure (HF)^15^. In the United States, AF is predicted to affect 6–12 million people by 2050 because of the aging population^16^. To the best of our knowledge, the association between RVO and AF has not been established previously in a longitudinal study with a large sample size. Therefore, in the current study, we investigated the risk of subsequent AF development following RVO using a representative nationwide sample of 1 million participants, which was provided by the National Health Insurance Service -National Sample Cohort from 2002–2013 (NHIS-NSC 2002–2013) database in South Korea.

## Results

### Patients’ baseline characteristics

Overall, 10,731 subjects, including 1,801 patients with RVO and 8,930 controls, were eligible for analysis (Table 1). During the entire study period (median, 7.7 years), AF occurred more frequently in subjects with RVO than in those without RVO (6.5% vs. 4.0%, p < 0.001). Comorbidities including HF, cerebrovascular disease, hypertension, diabetes mellitus (DM), chronic kidney disease (CKD), and liver disease were more common in the RVO group than in the sociodemographic-matched comparison group (p < 0.001, all). For sociodemographic variables, we found no difference in the proportion of patients according to the presence of RVO, which was used to perform matching between the two groups.

### Factors associated with AF occurrence

In multivariable model, we found that RVO was associated with a subsequent diagnosis of AF (hazard ratio [HR], 1.35; 95% confidence interval [CI], 1.09–1.67). HF (HR, 3.26; 95% CI, 2.65–4.02), hyperthyroidism (HR, 1.87; 95% CI, 1.09–3.19), acute myocardial infarction (HR, 1.55; 95% CI, 1.00–2.41), cerebrovascular disease (HR, 1.30; 95% CI, 1.03–1.64), and liver disease (HR, 1.24; 95% CI, 1.01–1.54) were associated with AF development in our multivariable model (Table 2); however, other comorbidities were not associated with AF development. Compared to male sex, female sex was more strongly associated with a decreased risk of AF occurrence (HR, 0.70; 95% CI, 0.58–0.84).

RVO was associated with a higher incidence of AF in both men and women (men: HR, 1.65; 95% CI, 1.22–2.24 vs. women: HR, 1.14; 95% CI, 0.85–1.54, Table 3). However, the HR was greater in men than in women, and in women, the result was not statistically significant. HF was the strongest predictive factor in both men and women (men: HR, 3.51; 95% CI, 2.58–4.78 vs. women: HR, 3.00; 95% CI, 2.26–3.98). In men, patients with hyperthyroidism (HR, 2.37; 95% CI, 0.96–5.86) and acute myocardial infarction (HR, 1.73; 95% CI, 0.94–3.16) were more likely to have an episode of AF, albeit marginally. In women, patients with cerebrovascular disease were more likely to have an episode of AF (HR, 1.37; 95% CI, 1.01–1.87).

**Figure 1:**
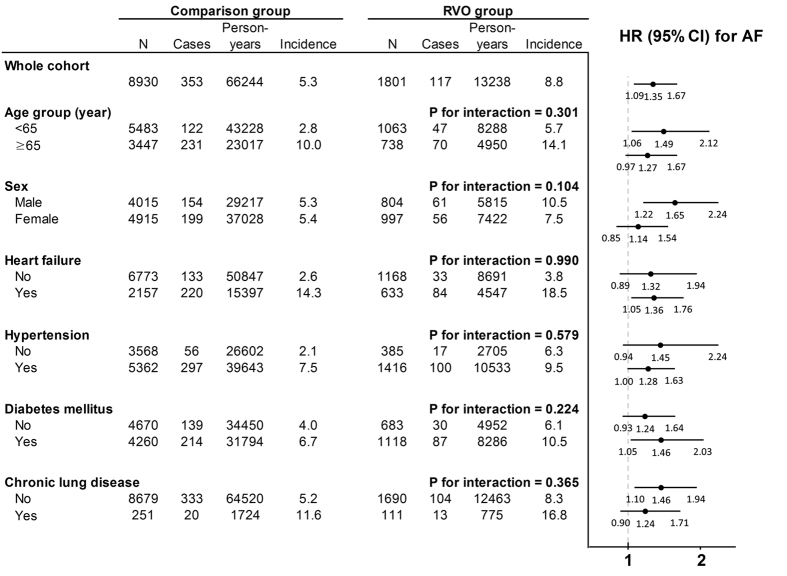
Incidence and risk of atrial fibrillation (AF) in the retinal vein occlusion (RVO) and comparison groups. Incidence rate per 1,000 person-years. CI, confidence interval; HR, hazard ratio. HRs were calculated based on multivariable Cox regression after being adjusted for sociodemographic factors and comorbidities. The P for interaction was calculated using the interaction term for RVO and each subgroup based on the multivariable Cox regression.

The follow-up period of 79,482 person-years, including 66,244 person-years for the comparison group and 13,238 person-years for the RVO group, was analyzed (Fig. 1). AF occurred in 8.8 per 1,000 person-years in the RVO group, and in 5.3 per 1,000 person-years in the comparison group. The incidence and risk of AF according to the presence of RVO were examined in each age and sex subgroups, and in the subgroups with or without comorbidities using multivariable Cox regression analysis. In the age subgroup analysis, subjects with RVO had a greater risk of subsequent AF in both age groups, albeit marginally in the older age group (age group <65 years: HR, 1.49; 95% CI, 1.06–2.12 vs. age group ≥65 years: HR, 1.27; 95% CI, 0.97–1.67). In men, RVO increased the risk of subsequent AF by an HR of 1.65 (95% CI, 1.22–2.24), whereas in women, the increased risk did not reach statistical significance (HR, 1.14; 95% CI, 0.85–1.54). RVO had an independent predictive value for AF development among subjects with HF (HR, 1.36; 95% CI, 1.05–1.76). However, in subjects without HF, the AF occurrence rate was relatively low (comparison group: 2.6 vs. RVO group: 3.8 per 1,000 person-years); thus, the difference was not statistically significant considering the large CI. In addition, in subjects with and without hypertension, DM, or chronic lung disease, RVO also increased the AF risk with HRs of >1; however, in the subgroups of subjects without hypertension or DM but with chronic lung disease, the results did not reach statistical significance due to the wide CIs.

### Survival curves for AF

**Figure 2:**
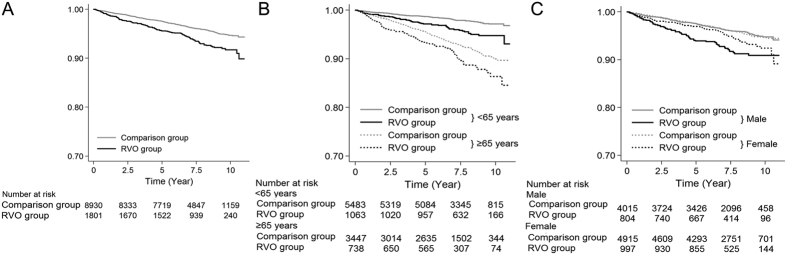
Kaplan-Meier survival curves for atrial fibrillation (AF) development in the retinal vein occlusion (RVO) and comparison groups during the study period. The cumulative AF-free survival curve (**A**) and AF-free survival curves grouped by age (**B**) and sex (**C**).

Figure 2 displays the Kaplan-Meier survival curves for up to 11 years. Overall, compared to patients in the comparison group, those with RVO experienced AF more frequently (Fig. 2A). The AF-free survival rates were different between the two groups regardless of the age subgroup (Fig. 2B). In terms of sex, the AF-free survival rate decreased faster starting early in the study period in men with RVO compared to men without RVO; this trend was not found in women (Fig. 2C).

## Discussion

We found an temporal relationship between newly developed RVO and subsequent AF development.

Recently, the retinal vasculature has gained more attention, because it is unique in that the retinal artery or vein and abnormal vasculature such as hypertensive retinopathy can be directly visualized using ophthalmoscopy. Therefore, RVO could be a surrogate marker for cardiovascular disease such as hypertension or stroke^12^. Although a new onset of RVO mainly causes decreasing vision, peripheral RVO does not affect vision in some cases. Therefore, the patient may not know about an RVO occurrence in his or her eyes. Underdiagnosed RVO could be detected by an ophthalmologist through the diagnosis of an eye complication such as sclerotic vessel change, optociliary shunt vessels (collaterals), sequelae of neovascularization, or thinning of the retinal nerve fiber layer^17^,^18^,^19^,^20^. The evaluation of these signs based on a retinal examination *in vivo* could be an easy, non-invasive way to stratify the risk of cardiovascular/cerebrovascular disease.

The characteristics of RVO are similar to those of stroke, because the structure of the retina is derived from the central nervous system^21^. Our previous study showed that RVO significantly increases the risk of stroke, with an HR of 1.48 (95% CI, 1.24–1.76) after adjusting for comorbidities^12^. AF has been the most common risk factor associated with stoke^22^. One case-control study showed that AF was more common in a CRVO group; however, cases of AF were excluded from the final model of stepwise logistic regression analysis^23^. According to a nationwide cohort study in Denmark of 605 patients with RVO retinal vascular (artery or vein) occlusion among 86,572 patients with non-valvular AF, RVO significantly increased the risk of stroke among patients with AF (HR, 1.26; 95% CI, 1.02–1.54)^24^. In this study, retinal vein or artery occlusion was suggested to be an independent risk factor for stroke in AF; therefore, it would merit at least 1 or 2 points in the CHA_2_DS_2_-VASc scoring system, fulfilling the stroke/thromboembolism criterion or vascular criterion^24^. In our study, RVO increased the likelihood of AF. Finally, RVO alone was independently associated with an increased risk of stroke and AF, and RVO increased the stroke risk in patients with AF. However, the effect size of the HRs could not be compared directly between studies because of the different ethnic populations and study designs.

The present study showed that the overall AF development was more common in men (Table 2), which is consistent with the results of previous reports^25^,^26^,^27^. Nevertheless, HF is one of the strongest predictors for incident AF in men (HR, 3.51) and women (HR, 3.00). However, other risk factors showed sex-related differences in terms of the AF risk. In a previous population-based study using a Danish cohort, hyperthyroidism was more common in women. However, male sex was associated with AF in those with hyperthyroidism^28^, and this significant result was consistent with our results. In a previous study using the Canadian Registry of AF, myocardial infarction was more prevalent in men, whereas stroke was more prevalent in women at the time of the initial presentation of AF^29^. In the present study, the HR for AF due to acute myocardial infarction was also greater in men than in women, and the HR for AF due to cerebrovascular disease was greater in women than in men. Results of previous studies and our study all indicate that there are sex-related differences in the clinical burden of AF. In particular, RVO also showed a sex-dependent association: RVO significantly increased the incidence AF in men, not in women (Table 3). Figure 2C also shows the different trends in the AF risk between men and women over time. The relatively large gap in the AF incidence between the two groups in men was sustained from the beginning to the end of the study period. However, the gap was relatively small in women. In our previous studies, RVO was a stronger predictor for acute myocardial infarction in younger men rather than in younger women^14^. In addition, RVO was a stronger predictor of HF in men than in women^13^. Sex-related differences have been recognized thus far, and RVO could be a possible cause of AF, acute myocardial infarction^14^, and HF^13^, particularly in men, as well as a cause of stroke in men and women^12^.

In accordance with previously published reports, we found that HF^30^, hyperthyroidism^28^, acute myocardial infarction, cerebrovascular disease, and liver disease^31^ were predictors of incident AF^32^. However, other comorbidities such as hypertension, DM, CKD, and chronic lung disease were not associated with AF based on our multivariable model. In the stratified analysis by HF (Fig. 1), RVO increased the risk of AF with an HR of approximately 1.3 in both subjects with or without HF. These results suggest an independent predictive value for RVO as well as HF for AF. Of note, RVO could be a better and earlier stage predictor for AF compared to other cardiovascular risk factors including hypertension, DM, or CKD.

Retinal microcirculation can be assessed non-invasively, directly, and safely. Retinopathy has been assumed to be a surrogate marker for microvascular dysfunction, which plays a pivotal role in the development of metabolic syndrome and cardiovascular disease^33^,^34^,^35^. Recently, metabolic syndrome has been assumed to be an important risk factor for AF development^36^,^37^. Autonomic neuropathy, oxidative stress, myocardial fibrosis, and microvascular dysfunction could be the underlying pathophysiologic mechanism for this association^38^,^39^. Further research to determine the causality and underlying mechanisms associated with the occurrence of AF and RVO is warranted. This epidemiological study does not provide evidence for the mechanism of RVO and AF.

The strengths of the present study include the large number of subjects and a long-term follow-up. Previous studies have shown an association between RVO and other cardio/cerebrovascular diseases such as stroke, hypertension, and DM. However, there have been no previous reports about the association between RVO and AF. This is the first report to examine AF development following RVO.

The most major limitation of the present study was the potential inaccuracy of the KCD code-diagnoses of RVO and AF. The accuracy of the health insurance service claims regarding RVO were mentioned in our previous report based on the NHIS-NSC 2002–2009 database^12^. The use of medical insurance claims dataset and the accuracy of these dataset are relevant issues to consider in the clinical setting. Unfortunately, there were no previous studies about the validity of national insurance claims for AF in South Korea. However, in Fig. 2A, the incidence of AF in the comparison group consistently increased for 11 years; this may partially represent the validity of AF diagnosis in our study. In addition, due to their asymptomatic nature, nasal occlusions are less likely to be diagnosed and reported. However, if patients with nasal-side RVO belong to the comparison cohort, the real HR may be greater than the current HR, as RVO increased the risk of AF.

Aside from the aforementioned possible inaccuracy of the KCD code-based diagnosis of RVO and AF, the following limitations of this study should be mentioned: (1) the possible misclassification of diagnoses for RVO, AF, or comorbidities; (2) possible underreporting of asymptomatic RVO or paroxysmal AF; (3) possibility of the delayed diagnosis of RVO or AF due to delayed visits to physicians; (4) possibility of inappropriately included patients with chronic RVO or AF; (5) potential that other health behavioral information such as alcohol use is missing; (6) higher possibility of bias among control patients from the medical claims than among control patients from the general population who have not received medical care; and (7) possible ethnic differences that may exist in ethnic groups other than the South Korean population that were not considered in this study.

In summary, RVO was associated with subsequent AF development in our multivariable Cox model. A retinal examination may be a safe method to evaluate one’s risk of AF. Therefore, ophthalmologists and cardiologists should be cautious of patients with RVO, as these patients, especially in men, should undergo cardiovascular risk screening to check for these disorders. These findings are limited, and they need to be confirmed by other observational studies.

## Methods

### Ethic statement

The institutional review board (IRB) of Severance Hospital at Yonsei University College of Medicine in Seoul, Korea approved this retrospective cohort study. The IRB waived the requirement to obtain informed consent, and this study was conducted in accordance with the tenets of the Declaration of Helsinki.

### Data source

This retrospective cohort study used the NHIS-NSC 2002–2013 dataset, comprising a random sample of 1,025,340 subjects, which amounted to approximately 2.2% of the entire population in the Korean NHIS in 2002. Data were produced by the NHIS using a systematic sampling method for the purpose of research. Random sampling was used based on 1,476 strata. As all individuals in South Korea are enrolled in the NHIS, All available data in the NHIS are centralized and include complete information, including diagnostic codes, procedures, prescription drugs used, and personal information for inpatient and outpatient visits. Moreover, diagnostic codes based on the KCD have been used in South Korea, and they are comparable to the International Classification of Diseases (ICD).

### Study population

The inclusion criteria were as follows: (1) patients in the study cohort who received medical care between 1st January 2003 and 31th December 2007 with a newly diagnosed RVO (KCD code H34.8, corresponding to ICD-9-CM codes 362.35, CRVO or 362.36, venous tributary [branch] occlusion); (2) subjects (comparison cohort) extracted using propensity-score matching (5 per each patient with RVO) from the database of 1 million subjects without RVO between January 2003 and December 2007; and (3) patients newly diagnosed as having AF (KCD code I48, corresponding to ICD-9-CM code 427.3, AF and flutter) from 2003–2013 to assess the outcome variable, AF occurrence. The exclusion criteria were as follows: (1) patients with chronic RVO before 2003; (2) patients with chronic AF before 2003; and (3) patients with AF occurrence before the occurrence of RVO, based on the patient’s visit date.

The variables for matching were as follows: (1) age (<50/50–59/60–69/70–79/≥80 years), (2) sex, (3) residential area, (4) household income (≤30%, 30–70%, and ≥70% of the median), and (5) year of enrollment (2003–2007). Comorbidities such as HF, hyperthyroidism, acute myocardial infarction, cerebrovascular disease, hypertension, DM, CKD, chronic lung disease, and liver disease were diagnosed based on the KCD, and they were defined as any diagnoses made from 2002–2007 (Supplementary Table 1).

### Statistical analysis

Characteristics of the study population were presented with descriptive statistics. We matched the RVO and comparison groups based on propensity score matching; the propensity scores were estimated by logistic regression analysis to predict RVO occurrence, and we controlled for age, sex, residency, income, and year of enrollment. Matching was performed using the greedy 8→1 digit-matching macro with a propensity score. Once a match was made, the match was not considered again. From the preliminary analysis based on the formula by Schoenfeld^40^, we could achieve more than 80% power with a sample size of 10,000 (ratio of cases to controls, 1:5) and an AF incidence of 4% for detecting an HR of 1.32 or greater. Each patient was tracked for up to 11 years from the first visitation date with a diagnosis of RVO (RVO group) or a randomly selected visitation date in the matched year (control group) to the last follow-up date in 2013 or to the date of the first diagnosis of AF. The overall AF survival rate was described using a Kaplan-Meier survival curve from 2003–2013 (median, 7.7 years). An association between RVO and the prospective risk of AF development was evaluated using Cox proportional hazard model. HRs with 95% CIs were calculated using a multivariable model. For subgroup analyses, the groups were stratified according to age, sex, and the presence of comorbidities. As the proportions of heart failure (26.0%), hypertension (42.7%), DM (20.7%), and chronic lung disease (30.1%) were relatively high (>20%) in the entire cohort, these comorbidities were used for subgroup analyses; the groups were stratified into patients with heart failure, hypertension, DM, or chronic lung disease and those without these comorbidities. A significance level of α 5% was considered statistically significant. Stata/MP2, version 14.0 (StataCorp, College Station, TX, USA) and the SAS System for Windows, version 9.4 (SAS Institute Inc., Cary, NC, USA) were used to perform the analyses.

## Additional Information

**How to cite this article**: Rim, T. H. *et al*. Evaluation of the Association Between Retinal Vein Occlusion and the Risk of Atrial Fibrillation Development: A 12-Year, Retrospective Nationwide Cohort Study. *Sci. Rep.* **6**, 34708; doi: 10.1038/srep34708 (2016).

**Publisher’s note**: Springer Nature remains neutral with regard to jurisdictional claims in published maps and institutional affiliations.

## Supplementary Material

Supplementary Information

## Figures and Tables

**Table 1:** Characteristics of the study population (comparison group [n = 8,930] and retinal vein occlusion [RVO] group [n = 1,801]).

Variables	Comparison group (column %)	RVO group (column %)	P value
Atrial fibrillation			<0.001
No	8,577 (96.1)	1684 (93.5)	
Yes	353 (4.0)	117 (6.5)	
Co-morbidities			
Heart failure	2,157 (24.2)	633 (35.2)	<0.001
Hyperthyroidism	115 (1.3)	31 (1.7)	0.147
Acute Myocardial Infarction	124 (1.4)	32 (1.8)	0.209
Cerebrovascular disease	815 (9.1)	245 (13.6)	<0.001
Hypertension	3,499 (39.2)	1082 (60.1)	<0.001
Diabetes mellitus	1,686 (18.9)	534 (29.7)	<0.001
Chronic kidney disease	251 (2.8)	111 (6.2)	<0.001
Chronic lung disease	2,651 (29.7)	574 (31.9)	0.065
Liver disease	1,526 (17.1)	406 (22.5)	<0.001
Variables for matching			
Year			
2003	1,786 (20.0)	362 (20.1)	0.991
2004	1,972 (22.1)	396 (22.0)	
2005	1,666 (18.7)	328 (18.2)	
2006	1,622 (18.2)	328 (18.2)	
2007	1,884 (21.1)	387 (21.5)	
Age group (year)			0.998
<50	1,841 (20.6)	370 (20.5)	
50~59	2,001 (22.4)	401 (22.3)	
60~69	3,026 (33.9)	609 (33.8)	
70~79	1,637 (18.3)	332 (18.4)	
≥80	425 (4.8)	89 (4.9)	
Sex			0.804
Male	4,015 (45.0)	804 (44.6)	
Female	4,915 (55.0)	997 (55.4)	
Residence			0.963
Seoul (metropolitan)	1,846 (20.7)	372 (20.7)	
2nd area	1,588 (17.8)	329 (18.3)	
3rd area	1,992 (22.3)	402 (22.3)	
4th area	3,504 (39.2)	698 (38.8)	
Household income			0.915
0~30%	2,062 (23.1)	409 (22.7)	
30–70%	2,952 (33.1)	603 (33.5)	
70–100%	3,916 (43.9)	789 (43.8)	

Seoul, a metropolitan area in Korea; 2nd area included the largest province; 3rd area included the 2nd largest city and the 2nd and 3rd largest provinces; 4th area included other areas.

**Table 2:** Results of the multivariable Cox regression analysis for the overall incidence of atrial fibrillation (n = 10,731).

Variables	Multivariable cox		
	adjusted HR	95% CI	p-value
Group			
Comparison group	1 (ref)		
RVO group	1.35	1.09–1.67	0.006
Comorbidities (reference group: subjects without each comorbidity)			
Heart failure	3.26	2.65–4.02	<0.001
Hyperthyroidism	1.87	1.09–3.19	0.022
Acute Myocardial Infarction	1.55	1.00–2.41	0.049
Cerebrovascular disease	1.30	1.03–1.64	0.027
Hypertension	1.19	0.95–1.49	0.121
Diabetes mellitus	1.13	0.92–1.38	0.247
Chronic kidney disease	1.03	0.71–1.48	0.876
Chronic lung disease	1.17	0.97–1.41	0.102
Liver disease	1.24	1.01–1.54	0.044
Age group (year)			
<50	1 (ref)		
50~59	2.56	1.49–4.39	0.001
60~69	3.56	2.13–5.97	<0.001
70~79	6.65	3.94–11.24	<0.001
≥80	7.05	3.86–12.89	<0.001
Sex			
Male	1 (ref)		
Female	0.70	0.58–0.84	<0.001
Residence			
Seoul (metropolitan)	1 (ref)		
2nd area	1.14	0.86–1.52	0.362
3rd area	0.94	0.71–1.25	0.681
4th area	1.01	0.79–1.30	0.911
Household income			
0~30%	1 (ref)		
30–70%	1.07	0.83–1.38	0.602
70–100%	0.97	0.76–1.22	0.773

CI, confidence interval; RVO, retinal vein occlusion; HR, hazard ratio. Seoul, a metropolitan area in Korea; 2nd area included the largest province; 3rd area included the 2nd largest city and the 2nd and 3rd largest provinces; 4th area included other areas.

**Table 3:** Results of multivariable Cox regression analysis after adjusting for sociodemographic factors for the overall incidence of atrial fibrillation according to sex.

Variables	Men (n = 4,819)			Women (n = 5,912)		
	adjusted HR	(95% CI)	p-value	adjusted HR	(95% CI)	p-value
Group						
Comparison group	1(ref)			1(ref)		
RVO group	1.65	1.22–2.24	0.001	1.14	0.85–1.54	0.388
Comorbidities (reference group: subjects without each comorbidity)						
Heart failure	3.51	2.58–4.78	<0.001	3.00	2.26–3.98	<0.001
Hyperthyroidism	2.37	0.96–5.86	0.062	1.69	0.87–3.31	0.123
Acute Myocardial Infarction	1.73	0.94–3.16	0.077	1.50	0.78–2.87	0.224
Cerebrovascular disease	1.19	0.83–1.69	0.344	1.37	1.01–1.87	0.044
Hypertension	1.18	0.85–1.63	0.322	1.21	0.89–1.64	0.228
Diabetes mellitus	1.12	0.82–1.52	0.474	1.12	0.85–1.47	0.420
Chronic kidney disease	0.91	0.54–1.52	0.721	1.15	0.68–1.94	0.600
Chronic lung disease	1.14	0.86–1.51	0.370	1.20	0.93–1.55	0.152
Liver disease	1.16	0.86–1.58	0.330	1.32	0.98–1.77	0.068

CI, confidence interval; RVO, retinal vein occlusion; HR, hazard ratio.
