# Supplementary material for: Evaluation of the Association Between Retinal Vein Occlusion and the Risk of Atrial Fibrillation Development: A 12-Year, Retrospective Nationwide Cohort Study
Source: Sci Rep. 2016 Nov 7;6:34708. doi: 10.1038/srep34708 (PMC5098134; doi:10.1038/srep34708)
Supplement: Supplementary Information [file srep34708-s1.pdf]

Evaluation of the Association Between Retinal Vein Occlusion and the Risk of Atrial Fibrillation Development: A 12-Year, Retrospective Nationwide Cohort Study

Tyler Hyungtaek Rim[1], Jaewon Oh[2], Christopher Seungkyu Lee[3], Sung Chul Lee[3], Seok-Min Kang[2], and Sung Soo Kim[3,4,5*]*

[1]Department of Ophthalmology, National Health Insurance Service Ilsan Hospital; [2]Division of Cardiology, Severance Cardiovascular Hospital and Cardiovascular Research Institute, Yonsei University College of Medicine, Seoul, Korea; [3]Department of Ophthalmology, Severance Hospital, Institute of Vision Research, Yonsei University College of Medicine, Seoul, Korea; [4]Yonsei Healthcare Big Data Based Knowledge Integration System Research Center, Yonsei University College of Medicine, Seoul, Korea; [5]Institute of Convergence Science, Yonsei University College of Medicine, Seoul, Korea

Supplementary Table 1. Korean Standard Classification of Disease (KCD) for comorbidities and International Classification of Disease 9th clinical modification (ICD-9-CM).

Comorbidities	KCD	ICD-9-CM
**Congestive heart failure**	I11.0, I13.0, I13.2, I25.5, I42.x, I43.x, I50.x	402, 404, 414.9, 425, 428
**Hyperthyroidism**	E05	242
**Myocardial infarction**	I21.x, I22.x , I25.2	410, 411
**Cerebrovascular disease**	I60.x, I61.x, I62.x, I63.x, I64.x, I65.x, I66.x, I67.x, I68.x, I69.x	430, 431, 432, 433, 434, 435, 436, 437, 438
**Hypertension**	I10	401
**Diabetes mellitus**	E10, E11, E12, E13, E14	250
**Chronic kidney disease**	N18.x, N19.x	585, 586
**Chronic lung disease**	I27.8, I27.9, J40.x, J41.x, J42.x, J43.x, J44.x, J45.x, J46.x, J47.x, J60.x, J61.x, J62.x, J63.x, J64.x, J65.x, J66.x, J67.x, J68.4, J70.1, J70.3	416, 491, 492, 493, 494, 495, 496, 500, 501, 502, 503, 504, 505, 506, 507, 508
**Liver disease**	B18.x, K70.x, K71.x, K72.x, K73.x, K74.x, K76.x, Z94.4, I85.0, I85.9, I86.4, I98.2	070, 571, 572, 573, 456.2
